# Comparative proteome analysis of *Saccharomyces cerevisiae*: A global overview of *in vivo* targets of the yeast activator protein 1

**DOI:** 10.1186/1471-2164-13-230

**Published:** 2012-06-09

**Authors:** He Jun, Thomas Kieselbach, Leif J Jönsson

**Affiliations:** 1 Department of Chemistry, Umeå University, SE-901 87, Umeå, Sweden; 2 Research Institute of Animal Nutrition, Sichuan Agricultural University, Ya’an, Sichuan, 625014, People’s Republic of China

**Keywords:** Yap1, *Saccharomyces cerevisiae*, Transcription factor, Stress response, Proteome

## Abstract

**Background:**

The activity of the yeast activator protein 1 (Yap1p) increases under stress conditions, which leads to enhanced transcription of a number of genes encoding protective enzymes or other proteins. To obtain a global overview of changes in expression of Yap1p-targeted proteins, we compared a Yap1p-overexpressing transformant with a control transformant by triplicate analysis of the proteome using two-dimensional gel electrophoresis (2-DE). Proteins of interest were identified using MALDI-MS or LC-MS/MS.

**Results:**

The relative quantities of 55 proteins were elevated significantly upon overexpression of Yap1p, and most of these proteins were found to have a Yap1p-binding site upstream of their coding sequences. Interestingly, the main metabolic enzymes in the glycolysis and pyruvate-ethanol pathways showed a significant increase in the Yap1p-overexpressing transformant. Moreover, a comparison of our proteome data with transcriptome data from the literature suggested which proteins were regulated at the level of the proteome, and which proteins were regulated at the level of the transcriptome. Eight proteins involved in stress response, including seven heat-shock and chaperone proteins, were significantly more abundant in the Yap1p-overexpressing transformant.

**Conclusions:**

We have investigated the general protein composition in Yap1p-overexpressing *S. cerevisiae* using proteomic techniques, and quantified the changes in the expression of the potential Yap1p-targeted proteins. Identification of the potential Yap1p targets and analysis of their role in cellular processes not only give a global overview of the ubiquitous cellular changes elicited by Yap1p, but also provide the framework for understanding the mechanisms behind Yap1p-regulated stress response in yeast.

## Background

The completion of the *Saccharomyces cerevisiae* genome project and molecular analysis of other fungal species has resulted in the identification of a growing number of yeast AP-1 transcription factors [1]. Characterization of these factors indicates that, like their mammalian counterparts, they activate gene expression in response to a variety of extracellular stimuli [1-4]. The *S. cerevisiae* transcription factor Yap1p belongs to the bZip (basic domain/leucine zipper) family of transcription factors that includes the yeast Gcn4p and the mammalian activator protein-1 proteins Fos and Jun [2]. Yap1p plays an important role in oxidative stress response and multidrug resistance by activating target genes encoding protective enzymes or other proteins [4-7]. These observations were corroborated by the analysis of yeast lacking specific Yap1 proteins and by the identification of genes with Yap1p-dependent expression [8-11]. More recently, we found that transcription of the *YAP1* gene in yeast was elevated in the presence of coniferyl aldehyde, an inhibitory compound derived from lignocellulose, and that overexpression of Yap1p in *S. cerevisiae* contributed to enhanced resistance against lignocellulose-derived inhibitory compounds and lignocellulosic hydrolysates [12,13]. However, the mechanisms behind Yap1p-regulated protective responses are still poorly understood.

Yap1p activates transcription by binding to specific DNA sequences located in the promoter of its target genes [1]. Currently, four predicted Yap1p-binding sites (TKACAAA, TGACTAA, TGACTCA, and TTACTAA) have been identified in hundreds of genes [14-16]. Obviously, the Yap1-regulated adaptation to various stimuli strongly depends on the expression of these target genes. To gain insights into how Yap1p regulates the protective response and how the yeast cell adapts to a changing environment, it is very important to get a global overview of changes in expression of these target genes [17].

DNA microarrays provide a practical and economical tool for studying expression of nearly every gene in yeast [18,19]. This approach can, in principle, be used to identify all the transcription targets of regulatory proteins like Yap1p. However, accumulating evidence indicates that mRNA abundance does not always correlate well with protein expression levels [19,20]. The present study was conducted to explore the changes in expression of Yap1p-targeted proteins at the proteome level. For this purpose, we utilized an *S. cerevisiae* transformant overexpressing Yap1p and performed triplicate analyses of the proteome by two-dimensional gel electrophoresis (2-DE). Proteins of interest were identified using mass spectrometry (MS). This study provides the mapping of the Yap1p-targeted proteins in *S. cerevisiae* and offers a global overview of the ubiquitous cellular changes elicited by overexpression of this important yeast transcription factor. To our knowledge this is the first report on the effect of Yap1 overexpression on the yeast proteome.

## Results

### Overexpression of Yap1p in *S. cerevisiae*

**Figure 1:**
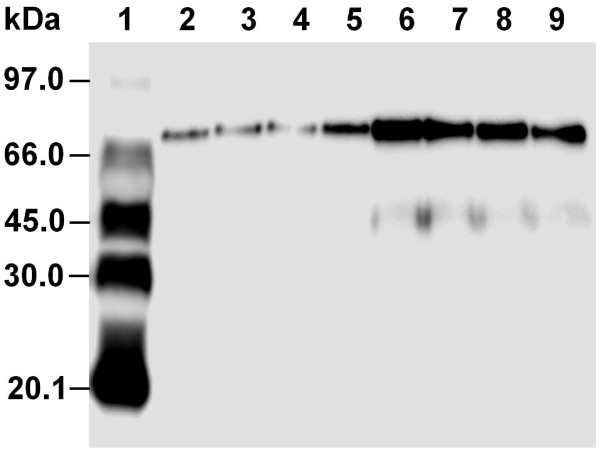
**Western-blot analysis of Yap1 protein from the control transformant and the Yap1p-overexpressing transformant.** Lane 1, protein marker; Lanes 2–5, four separate protein extracts from the control transformant; Lanes 6–9, four separate protein extracts from the Yap1p-overexpressing transformant.

To obtain a global overview of the *in vivo* Yap1p targets at the proteome level of *S. cerevisiae*, a comparative analysis was performed using a yeast transformant harboring a control plasmid and a transformant with a plasmid carrying the *YAP1* gene. Considering the possibility to control pH and maintain anaerobic conditions, yeast transformants were cultivated in a multi-bioreactor and the fermentation was discontinued when the cells were still in the exponential growth phase. Before 2-DE analysis, overexpression of Yap1p was validated by western-blot analysis. As expected, the Yap1 protein was present at elevated levels in the Yap1p-overexpressing transformant (Figure 1). The level was estimated to be approx. four-fold higher than in the control transformant.

### 2-DE analyses of protein extracts from *S. cerevisiae*

Yeast cells from both cultures were harvested and proteins were extracted using the extraction protocol developed by Kolkman et al. [21], which we further optimized for our yeast samples. In the protocol developed by Kolkman et al., the cells are lyophilized and subsequently vortexed with glass beads prior to boiling with SDS. In order to improve cell disruption, we introduced an additional step. Before the SDS boiling, the yeast cells were disrupted in extraction buffer containing thiourea. Cell debris which was not dissolved in the extraction buffer was further exposed to boiling with SDS. More high-molecular-mass proteins (> 70 kDa) were observed on the 2-D gels when this optimized extraction protocol was adopted.

**Figure 2:**
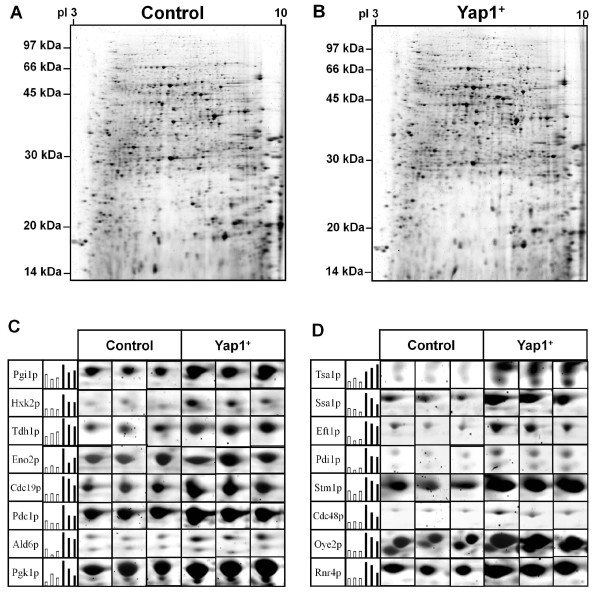
**Images of the 2-D gels of protein extracts from yeast cells.** The 2-D gels were loaded with 200 μg of protein extract from the control transformant (A) or the Yap1p-overexpressing transformant (B). Proteins were visualized by fluorescence staining (Sypro Ruby). Typical data from triplicate 2-D gel images are shown in C (proteins involved in carbon metabolism) and D (proteins involved in pathways other than carbon metabolism). Histograms show the protein abundance, with the spot volume of the 2-D gels indicated in white (control) and black (Yap1p overexpression).

In Figure 2, the 2-D gel electrophoresis images of the control transformant (Figure 2A) and the Yap1p-overexpressing transformant (Figure 2B) are shown. By using the SYPRO Ruby staining method, more than 2,000 protein spots were detected on each 2-D gel. This number is higher than what has been achieved by silver staining, for which only a few hundred spots were detected [21]. The 2-DE analyses were performed in triplicate to allow statistical analysis, and Student’s *t*-test was used to determine if the relative change in protein expression was statistically significant. Based on this analysis, protein spots that were significantly up-regulated upon Yap1p overexpression were identified on the 2-D gels. In total, 78 such spots were detected on the 2-D gels. Typical examples are shown in Figure 2C and D. These spots were further analyzed by MALDI-MS and LC-MS/MS, resulting in identification of 55 unique proteins (MALDI-MS was used for most of the protein spots (1-73), while LC-MS/MS was used for analysis of a few spots (74-78) for which MALDI-MS analysis did not give satisfactory results). Interestingly, some of the proteins were identified in more than one spot on the 2-D gels.

### Comparative proteome analysis of *S. cerevisiae*

**Figure 3:**
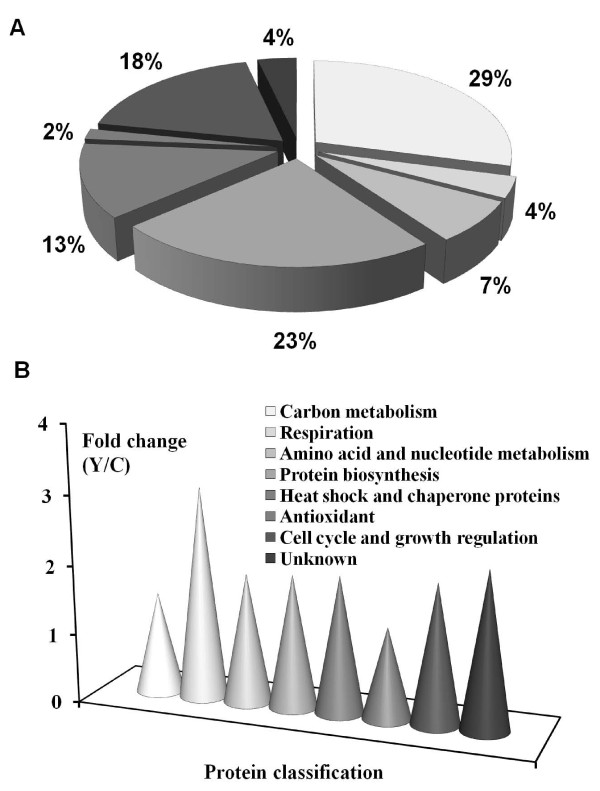
**Functional classification of 55 up-regulated proteins in** ***S. cerevisiae*** **upon Yap1p overexpression (A) and fold changes (Y/C) of combined spot volumes of all identified proteins (B).**

The 55 proteins that were identified are listed in Table 1 and the relative quantity is indicated. Of the averaged total spot volumes of the 55 identified proteins (Table 1), 16 changed significantly at 99% confidence level (*P* < 0.01), 33 changed significantly at the 95% confidence level (*P* < 0.05), and 6 changed significantly at 90% confidence level (*P* < 0.10). The identified proteins were divided into different categories, namely enzymes involved in carbon metabolism and proteins involved in pathways other than carbon metabolism, such as protein biosynthesis, cell cycle and growth regulation, etc. (Figure 3A). It is noteworthy that 16 proteins that play a role in carbon metabolism were up-regulated in the Yap1p-overexpressing yeast transformant. These proteins include ten glycolytic enzymes (Pgi1p, Hxk2p, Fba1p, Tdh1p, Tdh2p, Tdh3p, Pgk1p, Gpm1p, Eno2p, and Cdc19p), four enzymes involved in conversion of pyruvate to ethanol (Pdc1p, Adh1p, Ald6p, and Dld3p), and two enzymes (Tkl1p and Gnd1p) that are involved in the pentose phosphate pathway. Based on image analysis, we observed that the combined spot volumes of all identified enzymes involved in carbon metabolism enzymes increased about 1.5 fold in the Yap1p-overexpressing transformant (Figure 3B).

**Table 1:** **Relative changes in protein expression on overexpression of Yap1p.**^**1**^

**Protein**	**Description**	**ORF**	**Normalized averaged spot quantity**				**Y/C**	***p*-value**	**YBS**
			**Control**		**Yap1**^**+**^				
			**Avol**	**SD**	**Avol**	**SD**			
*Protein involved in carbon metabolism pathways*									
**Glycolysis**									
Fba1p^#^	Fructose-bisphosphate aldolase	YKL060C	3134	89	5986	1035	1.91	0.009	+
Pgi1p^#^	Glucose-6-phosphate isomerase	YBR196C	540	120	950	205	1.76	0.040	-
Hxk2p	Hexokinase-2	YGL253W	167	10	374	92	2.24	0.018	-
Tdh1p	Glyceraldehyde-3-phosphate dehydrogenase 1	YJL052W	709	148	1390	256	1.96	0.018	+
Tdh2p	Glyceraldehyde-3-phosphate dehydrogenase 2	YJR009C	1769	118	2516	539	1.42	0.079	-
Tdh3p	Glyceraldehyde-3-phosphate dehydrogenase 3	YGR192C	6899	401	9620	195	1.39	<0.001	+
Pgk1p^#^	Phosphoglycerate kinase	YCR012W	3970	506	5204	931	1.31	0.045	+
Gpm1p	Phosphoglycerate mutase 1	YKL152C	2960	508	4727	284	1.60	0.006	+
Eno2p^#^	Enolase 2	YHR174W	11810	323	15053	2147	1.27	0.041	+
Cdc19p^#^	Pyruvate kinase 1	YAL038W	1460	44	3155	568	2.16	0.007	+
**Pyruvate branchpoint**									
Pdc1p^#^	Pyruvate decarboxylase isozyme 1	YLR044C	7914	380	7443	1520	1.55	0.043	-
Adh1p	Alcohol dehydrogenase 1	YOL086C	4200	665	5158	743	1.31	0.041	+
Ald6p	Aldehyde dehydrogenase	YPL061W	161	33	329	40	2.04	0.005	+
Dld3p	D-lactate dehydrogenase 3	YEL071W	290	20	501	79	1.73	0.011	-
**Pentose phosphate pathway**									
Tkl1p^#^	Transketolase 1	YPR074C	284	10	619	165	2.18	0.025	+
Gnd1p	6-phosphogluconate dehydrogenase 1	YHR183W	633	124	876	116	1.37	0.075	-
*Protein involved in pathways other than carbon metabolism*									
**Amino-acid and nucleotide metabolism**									
Sah1p	Adenosylhomocysteinase	YER043C	631	143	1310	304	2.08	0.025	-
Shm2p	Serine hydroxymethyltransferase	YLR058C	486	26	944	176	1.94	0.011	-
Aro9p	Aromatic amino acid aminotransferase 2	YHR137W	116	19	164	13	1.41	0.022	-
Lys9p	Saccharopine dehydrogenase	YNR050C	147	13	364	123	2.48	0.039	+
**Protein biosynthesis**									
Eft1p^#^	Elongation factor 2	YOR133W	136	58	456	174	3.35	0.039	+
Yef3p	Elongation factor 3A	YLR249W	221	37	378	41	1.71	0.008	+
Gus1p	Glutamyl-tRNA synthetase	YGL245W	100	17	254	29	2.54	0.001	+
Tef4p	Elongation factor 1-gamma 2	YKL081W	846	20	1059	11	1.25	0.004	+
Sgt2p	Small glutamine-rich tetratricopeptide repeat-containing protein 2	YOR007C	588	67	855	101	1.45	0.019	+
Rps7ap	40 S ribosomal protein S7-A	YOR096W	1377	459	2742	136	1.99	0.008	+
Grs1p	Glycyl-tRNA synthetase 1	YBR121C	254	37	361	50	1.42	0.042	+
Kar2p	78 kDa glucose-regulated protein	YJL034W	97	17	303	100	3.13	0.024	-
Pdi1p^#^	Protein disulfide-isomerase	YCL043C	85	24	354	88	4.17	0.007	-
Rpl5p	60 S ribosomal protein L5	YPL131W	364	137	1298	329	3.56	0.066	-
Rpp0p	60 S acidic ribosomal protein P0	YLR340W	752	88	1153	161	1.53	0.019	-
Rps3p	40 S ribosomal protein S3	YNL178W	342	25	801	214	2.34	0.095	+
Pab1p^#^	Polyadenylate-binding protein	YER165W	301	52	484	63	1.61	0.018	+
**Heat-shock and chaperone proteins**									
Ssa1p	Heat shock protein	YAL005C	282	83	792	247	2.81	0.027	+
Ssa2p^#^	Heat shock protein	YLL024C	1279	211	2021	405	1.58	0.048	-
Ssb1p^#^	Heat shock protein	YDL229W	258	64	558	141	2.16	0.028	+
Ssb2p	Heat shock protein	YNL209W	295	72	578	130	1.96	0.031	+
Hsp82p	ATP-dependent molecular chaperone	YPL240C	77	17	253	55	3.30	0.006	+
Hsc82p^#^	ATP-dependent molecular chaperone	YMR186W	209	44	487	161	2.32	0.045	-
Sse1p	Heat shock protein homolog	YPL106C	73	16	194	68	2.66	0.041	+
**Antioxidants**									
Tsa1p	Peroxiredoxin TSA1	YML028W	656	99	909	90	1.39	0.031	+
**Respiration**									
Oye2p^#^	NADPH dehydrogenase 2	YHR179W	2129	174	6677	1357	3.14	0.005	+
Atp2p	ATP synthase subunit beta	YJR121W	158	13	313	35	1.98	0.002	+
**Cell cycle and growth regulation**									
Bfr1p	Nuclear segregation protein	YOR198C	228	79	558	227	2.53	0.066	+
Stm1p	Suppressor protein	YLR150W	2101	245	3565	444	1.70	0.007	+
Cdc48p^#^	Cell division control protein 48	YDL126C	121	8	319	110	2.65	0.036	+
Vma1p	V-type proton ATPase subunit A	YDL185W	220	49	318	26	1.44	0.039	-
Vma2p	V-type proton ATPase subunit B	YBR127C	233	99	412	85	1.77	0.076	-
Sis1p	Protein SIS1	YNL007C	314	186	702	79	2.24	0.029	+
Rnr4p	Ribonucleoside-diphosphate reductase small chain 2	YGR180C	623	19	1293	317	2.08	0.022	-
Bgl2p	Endo-beta-1,3-glucanase	YGR282C	580	45	3453	328	5.95	0.007	+
Bmh1p	Protein BMH1	YER177W	1119	141	1564	30	1.40	0.049	+
Srp1p	Importin subunit alpha	YNL189W	185	24	354	88	1.91	0.006	+
**Others**									
Ola1p	Uncharacterized GTP-binding protein	YBR025C	281	20	449	79	1.59	0.024	+
Ynn4p	Uncharacterized protein YNL134C	YNL134C	438	176	1171	354	2.67	0.033	+

^1^The normalized volume of spots from three replicate 2-D gels was averaged and the standard deviation was calculated for each yeast transformant. A Student’s *t*-test was performed to determine if the relative change was statistically significant. If proteins were identified and quantified in more than one spot on the 2-D gels, the total spot volume was calculated (indicated with “^#^”). YBS, nucleotide sequence with (+) or without (−) predicted Yap1p-binding site; Avol, averaged value from three spot volumes; Y/C, the relative change (Yap1p-overexpression *versus* control).

Eight proteins involved in stress response were identified that were significantly more abundant in the Yap1p-overexpressing transformant. These proteins include seven heat-shock and chaperone proteins (Ssa1p, Ssa2p, Ssb1p, Ssb2p, Hsp82p, Hsc82p, and Sse1p) and one peroxiredoxin (Tsa1p). Compared to the control transformant, most of the heat-shock and chaperone proteins showed more than 2-fold increase in the Yap1p-overexpressing transformant (Table 1). Moreover, 13 proteins involved in protein biosynthesis and 10 proteins involved in cell cycle and growth regulation were identified on the 2D gels. The combined spot volumes of all the identified proteins increased about two-fold (Figure 3B).

While the results reported were reproducibly obtained on all 2-D gels analyzed, it should be noted that some spots might have contained co-migrating proteins that were not detected in the MALDI-MS analysis. These proteins would have affected the relative quantification of the up-regulated proteins. As MALDI-MS identifies the prevalent proteins that are present in a gel sample, these errors are, however, considered to be negligible.

## Discussion

The yeast transcription factor Yap1p is crucial for the normal response of yeast cells to a variety of stress conditions including oxidative stress, drug-induced stress, and heat shock [1,2]. Previous studies indicated that most stress conditions induced the activity of Yap1p and, as a consequence, resulted in elevated expression of a number of genes encoding proteins that protect the cells against stress-induced damage [4-7]. Although, Yap1p-dependent expression of a diverse range of proteins is essential for viability, a major unresolved question concerns the complete pattern of proteins expressed in a cell upon Yap1p overexpression. We report here the first characterization of the proteome of Yap1p-overexpressing yeast. The experimental approach enables the analysis of the relative protein levels under conditions that mimic stress (Yap1p-overexpression). This resulted in many changes in the levels of proteins involved in crucial biological pathways.

**Figure 4:**
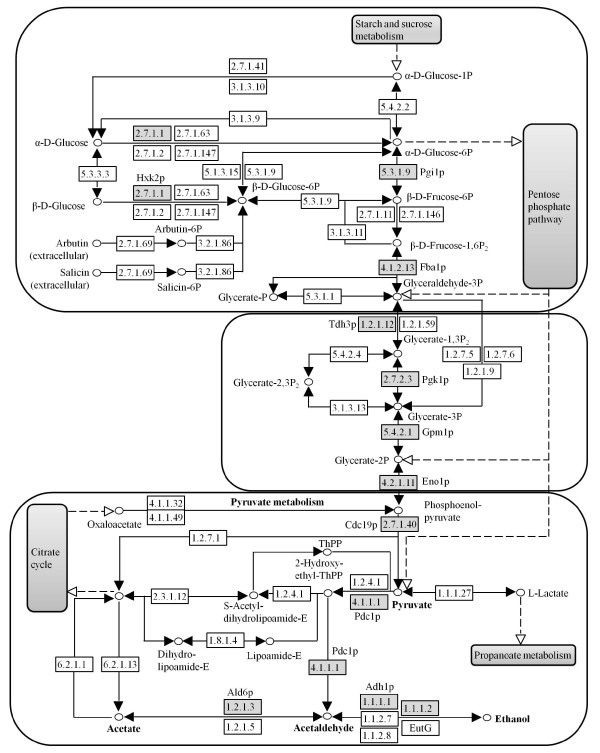
**Changes of the glycolysis and pyruvate-ethanol pathways in** ***S. cerevisiae*** **(items selected from** **http://www.genome.jp/kegg/pathway.html) upon Yap1p overexpression. Gray boxes indicate up-regulated proteins.**

The glycolytic pathway plays a fundamental role in the provision of metabolic energy and intermediates during fermentative growth of the yeast *S. cerevisiae*[22]. The glycolytic enzymes, which are involved in the conversion of glucose to pyruvate, were significantly more abundant in the Yap1p-overexpressing yeast (Figure 4). In particular, the relative abundance of Hxk2p and Cdc19p increased more than two-fold. This is most likely due to the fact that they are rate-limiting enzymes in glycolysis [22]. In *S. cerevisae*, the first irreversible step of glycolysis (phosphorylation of glucose) can be catalyzed by three enzymes, namely the hexokinases Hxk1p and Hxk2p and the glucokinase Glk1p [23]. However, Hxk2p appears to play the main role since it is the predominant isoenzyme during growth on glucose [23,24]. Moreover, Hxk2p has been identified in the nucleus of the cell and is required for glucose-induced repression of several genes including HXK1 and GLK1 [25,26]. Our results are consistent with these findings, since Hxk1p and Glk1p were not detected on the 2-D gels. Cdc19p (also known as Pyk1p), which catalyzes the final step of glycolysis, namely the conversion of phosphoenolpyruvate to pyruvate, is the main pyruvate kinase in the glycolysis pathway. In the present study, the relative abundance of Cdc19p increased more than two-fold in the Yap1p-overexpressing yeast. Another isoenzyme, Pyk2p (a second yeast pyruvate kinase), was, however, not detected on the 2D gels. The regulation mode of pyruvate kinases is similar to that of hexokinases since Cdc19p is tightly regulated and activated by fructose-1,6-bisphophate (FBP), whereas Pyk2p is subject to glucose repression and appears to be insensitive to FBP levels [27].

Relatively few of the identified proteins in the glycolysis and pyruvate-ethanol pathways exhibited more than two-fold increment in the Yap1p-overexpressing yeast. The response suggests that the levels are affected by Yap1p in different ways, and that other factors may also play a role in the regulation. Moreover, none of the enzymes in the citric acid cycle (TCA cycle) were found to be significantly up-regulated upon Yap1p-overexpression. This is probably a result of the anaerobic cultivation conditions. During alcoholic fermentation of sugars, the glycolytic genes are the most efficiently expressed genes in yeast, and glycolytic enzymes comprise over 30% of the soluble cell protein [28]. Moreover, two crucial enzymes (Pdc1p and Adh1p) involved in the pyruvate-ethanol pathway were significantly up-regulated in the Yap1p-overexpressing yeast, and that would probably result in a shortage of substrate (pyruvate) for the TCA cycle. An alternative mode of glucose oxidation is offered by the pentose phosphate pathway, which provides the cell with pentose sugars and cytosolic NADPH, necessary for biosynthetic reactions, such as the production of fatty acids, amino acids, and sugar alcohols [29]. Importantly, the pathway is also necessary to protect yeast cells against oxidative stress, since NADPH is an essential cofactor for anti-oxidative enzymes [30]. In the present study, two proteins (Tkl1p and Gnd1p) involved in this pathway were identified on the 2-D gels as occurring at higher levels in Yap1p-overexpressing yeast (Table 1).

Overexpression of Yap1p in *S. cerevisae* resulted in up-regulation of a number of proteins involved in stress response, including seven heat-shock and chaperone proteins (Hsps), and one peroxiredoxin (Table 1). The expression of Hsps is one of the conserved mechanisms of cellular protection [31]. Expression of Hsps was first observed when fruit flies were exposed to high temperatures [32]. However, an elevation of temperature is not the only way to induce the expression of Hsps. Heavy metals, ethanol, oxygen radicals and peroxides are among a large group of agents that can induce Hsps [31-33]. Since stress response also induce the activity of Yap1p [1,34], our result suggests that Yap1p may be an important activator for Hsps when yeast cells are exposed to stress conditions. The peroxiredoxin Tsa1p was 1.4-fold up-regulated upon overexpression of Yap1p. Tsa1p belongs to a family of thiol-specific peroxidases that catalyze the reduction of peroxides through oxidation of Cys [35]. It has also been identified as the key peroxidase suppressing genome instability and protecting against cell death in yeast [36]. However, the up-regulation of Tsa1p was relatively modest (Table 1), and the role of Tsa1p in Yap1p-mediated stress response remains elusive. The number of identified antioxidant proteins was rather less than expected, since Yap1p has been described primarily as a central regulator of the response to oxidative stress in *S. cerevisiae*[5].

A number of proteins involved in cell cycle and growth regulation were identified on the 2-D gels. Interestingly, the protein with the highest absolute increase was the endo-beta-1,3-glucanase (Bgl2p), which is involved in yeast cell wall maintenance [37]. Another significantly up-regulated protein was the cell-division control protein 48 (Cdc48p), which is an abundant and evolutionarily conserved protein involved in many aspects of cellular activities, including homotypic membrane fusion of organelles, ERAD, ubiquitin/proteasome-mediated protein degradation, and cell-cycle control [38-40]. Interestingly, Cdc48p has been observed to participate in the maintenance of the yeast cell wall [41]. Yap1p-mediated up-regulation of Bgl2p and Cdc48p in yeast may be of great importance, since the cell wall gives the cell rigidity and strength, and offers protection against a variety of different forms of stress.

To investigate if the genes encoding these up-regulated proteins are potential transcription targets of Yap1p, we have searched upstream of each nucleotide sequence for the predicted Yap1p-binding sites [14-16]. As expected, most genes encoding the identified proteins were found to have a binding site in their promoter region (Table 1). This indicates that most of the up-regulated proteins are transcription targets of Yap1p. However, none of the four predicted binding sites were observed on the coding sequences of proteins such as the glycolytic enzymes Hxk2p, Pgi1p and Tdh2p, which suggests that their levels are affected by Yap1p in a different way.

**Figure 5:**
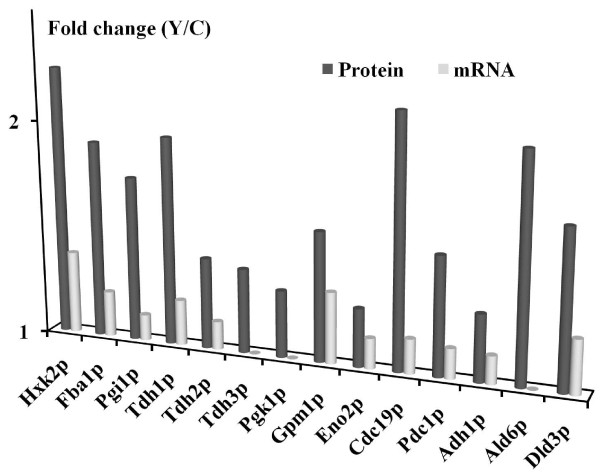
**Comparison of the proteome data (this work) and transcriptome data (ref.[17]) for selected proteins involved in the glycolysis and the pyruvate-ethanol pathways.**

Finally, we compared our proteome data with the literature data for changes of the transcriptome [17]. As shown in Figure 5, most glycolytic enzymes except for Tdh3p and Pgk1p were significantly up-regulated at both the mRNA and the protein level, which suggests that most enzymes in glycolysis are mainly regulated at the transcriptome level. In the pyruvate-to-ethanol pathway, Ald6p is most likely regulated at the level of the proteome, because only the proteome changes were significant, whereas Pdc1p and Adh1p are regulated transcriptionally, as both the mRNA and the protein levels were up-regulated in Yap1p-overexpressing yeast. Although, there are several minor differences between the two studies (i.e. cultivation conditions), it is still noteworthy that mRNA abundance does not always correlate well with protein expression levels. Compared with transcriptome studies, proteome studies are generally limited by the number of gene products that can be analyzed simultaneously [42]. In the present study, the total number of up-regulated targets upon Yap1p-overexpression is less than the number for corresponding transcriptome analysis [17]. Our results, however, not only show that there are some discrepancies between transcriptome and the proteome data, but also indicate that the combination of the two methodologies can potentially lead to a more complete understanding of the molecular biology of *S. cerevisiae*.

## Conclusions

We have investigated the general protein composition in Yap1p-overexpressing *S. cerevisiae* using proteomic techniques, and quantified the changes in the expression of the potential Yap1p-targeted proteins. Mapping the changes in protein expression levels provides insight on how *S. cerevisiae* adapts to a conventional stress condition resulting in activation of Yap1p. Moreover, we were able to elucidate if gene expression in the glycolytic and pyruvate-ethanol pathways are primarily regulated at the level of the proteome or of the transcriptome. Importantly, studies of Yap1p using different experimental conditions may help to further improve our understanding of its effect. Identification of the potential Yap1p-targeted proteins and their mapping into cellular processes not only give a global overview of the ubiquitous cellular changes elicited by Yap1p, but also provide the framework for understanding the mechanisms behind Yap1p-regulated protective response in yeast.

## Methods

### Transformants and preparation of inoculums

All yeast transformants (a Yap1p-overexpressing transformant and a control transformant) that were used in this study were previously constructed and stored in our laboratory [13]. Yeast transformants designated Y (with the *YAP1* gene under the control of *PGK1* promoter) and C (negative control carrying the same plasmid but without any *YAP1* gene) were streaked on SC–Ura agar plates (20 g/l glucose, 6.7 g/l yeast nitrogen base without amino acids, 10% amino-acid supplement solution × 10 excluding uracil, and 20 g/l agar) [43], which were then incubated at 30 °C for 72 h. Inoculum cultures of the two *S. cerevisiae* transformants were prepared in 500 ml shake flasks with 140 ml of SC-Ura medium (excluding the agar). The flasks were inoculated with cells from the agar plates and incubated for approximately 17 h at 30 °C with agitation (Infors Ecotron, Infors AG, Bottmingen, Switzerland). The cells were harvested in the exponential growth phase by centrifugation at 1,200 × *g* for 10 min at 4 °C (Allegra X-22R, Beckman Coulter, Brea, CA, USA). The cells were then resuspended in a suitable amount of sterile H_2_O to yield an inoculum of 0.1 g/l (DW) in all bioreactor vessels.

### Yeast fermentation in multi-bioreactor

The cultivation of the two transformants Y and C was carried out with a multi-bioreactor system (Sixfors from Infors AG, Bottmingen, Switzerland). Four 350-ml-bioreactor vessels (two vessels per transformant) equipped with condensers, FermProbe pH-electrodes (Broadley James Corporation, Irvine, CA) and OxyProbe polarographic dissolved-oxygen sensors (pO2-electrodes) (Broadley James Corporation) were sterilized through autoclavation and filled with 250 ml modified SC–Ura medium. The composition of the medium was: 40 g/l glucose, 13.4 g/l yeast nitrogen base without amino acids, 10% amino-acid supplement solution × 10 excluding uracil, 0.1% of an ergosterol/Tween 80 mixture (consisting of 10 g/l ergosterol and 420 g/l Tween 80), and 8 drops of antifoam.^12^ The pH electrodes and the pO2 electrodes were calibrated prior to start-up. Two ml of inoculum were added to each bioreactor vessel to an initial biomass concentration of 0.1 g/l (DW). Throughout the fermentation, the temperature was kept at 30 °C, the stirring was kept at 300 rpm, and the pH was kept at 5.5 by automatic addition of 0.5 M NaOH. Nitrogen gas (15 l/h) was used to maintain anaerobic conditions. The fermentation was discontinued after seven hours when the cells were in the exponential growth phase and had reached a cell density of 1 g/l (DW). The yeast cells were harvested by centrifugation at 3,000 × *g* for 5 min at 4 °C, and stored at −80 °C before protein extraction.

### Protein extraction and purification

Yeast protein extracts were prepared for analysis with 2-DE using a modified approach of Kolkman et al. [21]. In brief, about 100 mg of lyophilized cell pellet were resuspended in 600 μl extraction buffer [7 M urea, 2 M thiourea, 4% (w/v) CHAPS (3-[(3-cholamidopropyl) dimethylammonio]-1-propanesulfonate), 40 mM dithiothreitol]. Protease inhibitor cocktail (Calbiochem, La Jolla, CA) and glass beads (acid washed, 425–600 μm, Sigma-Aldrich, St. Louis, MO) were added to the cell suspension. Cells were disrupted by vortexing six times 60 s (the samples were cooled on ice for 30 s in between the vortex steps). The cell extract was transferred to a fresh tube and centrifuged at 20,000 × *g* for 10 min at 4 °C. The supernatant was transferred completely to a fresh microcentrifuge tube and recovered as Fraction 1. The insoluble fractions were suspended in 400 μl SDS-buffer (2% SDS, 40 mM Tris, 60 mM DTT) by thorough vortexing and pipetting up and down with a 200 μl pipette tip for 10 times. The sample was boiled for 10 min and subsequently cooled on ice. After centrifugation for 10 min (20,000 × *g*, 4 °C), the supernatant (Fraction 2) was then transferred to a fresh microcentrifuge tube and mixed with Fraction 1. Subsequently, 75 μl of a DNase and RNase solution [1% (w/v) DNase I, 0.25% (w/v) RNase A, 50 mM MgCl_2_, 0.5 M Tris–HCl, pH 7.0] were added and the combined fractions were incubated on ice. The mixed protein extract was then purified by using a 2-D Clean-Up Kit (GE Healthcare, Uppsala, Sweden), and the purified protein sample was dissolved in rehydration solution [7 M urea, 2 M thiourea, 4% (w/v) CHAPS, 0.002% (w/v) bromophenol blue] supplemented with 2% (v/v) 3–10 NL IPG buffer (GE Healthcare) and 5.4 mg/ml dithiothreitol. Total protein concentration was determined using the 2-D Quant Kit (GE Healthcare). Aliquots of extracellular protein samples were stored at −80 °C before proteomic assays.

The peaklist files of the processed mass spectra of spots 1 to 78 are provided in the appendix. Additional file 1: Spots 1 to 73 were analysed by MALDI-TOF-MS and the peaklist files are available in the supplementary zip files: Additional file 2: 1-5_MALDI_MS_data, Additional file 3: 6-10_MALDI_MS_data, Additional file 4: 11-15_MALDI_MS_data, Additional file 5: 16-20_MALDI_MS_data, Additional file 6: 21-25_MALDI_MS_data, Additional file 7: 26-30_MALDI_MS_data, Additional file 8: 31-35_MALDI_MS_data, Additional file 9: 36-40_MALDI_MS_data, Additional file 10: 41-45_MALDI_MS_data, Additional file 11: 46-50_MALDI_MS_data, Additional file 12: 51-55_MALDI_MS_data, Additional file 13: 56-60_MALDI_MS_data, Additional file 14: 61-65_MALDI_MS_data, Additional file 15: 66-69_MALDI_MS_data,_and Additional file 16: 70-73_MALDI_MS_data. As for spots 74 to 78, analysis was performed by LC-MS/MS and the corresponding mzML files are provided in the supplementary zip files Additional file 17: 74_LC_MS_MS_data, Additional file 18: 75_LC_MS_MS_data, Additional file 19: 76_LC_MS_MS_data, Additional file 20: 77_LC_MS_MS_data, and Additional file 21: 78_LC_MS_MS_data.

### Western-blot analysis of Yap1 protein

The crude protein extracts were separated by SDS-PAGE after adding 5× Laemmli sample buffer and boiling. The separated proteins were transferred onto a PVDF membrane by semi-dry blotting and probed (1:2000 dilution, incubated overnight at 4 °C) with a rabbit polyclonal antibody directed against amino-acid residues 351–650 at the C-terminus of *S. cerevisiae* Yap1p (Santa Cruz Biotechnology, Santa Cruz, CA). Goat anti-rabbit IgG-HRP (Santa Cruz Biotechnology) was used as secondary antibody. Bound antibodies were detected by the ECL Prime western blotting detection reagent (GE Healthcare) using a CCD-based imager (ImageQuant LAS 4000, GE Healthcare).

### 2-D gel electrophoresis

For the first dimension (IEF), an amount of 200 μg of protein (300 μl) prepared as described in section Protein Extraction and Purification was loaded on a 13 cm Immobiline Dry-Strip pH 3–10 NL (GE Healthcare), and the IPG strips were rehydrated overnight at room temperature. Isoelectric focusing (IEF) was performed with a Multiphor II system (GE Healthcare) at 20 °C with a 3-phase gradient program: 500 V for 0.25 kVh, 3500 V for 5.25 kVh, and 3500 V for 45 kVh. Prior to the second dimension (SDS-PAGE), the IPG strips were incubated for 15 min in equilibration buffer [50 mM Tris–HCl, 6 M urea, 30% (v/v) glycerol, 2% (w/v) SDS, 0.002% (w/v) bromophenol blue] containing 1% (w/v) dithiothreitol, followed by 15 min incubation in equilibration buffer containing 2.5% (w/v) iodoacetamide. Second-dimension electrophoresis was performed on PROTEIN^TM^ II electrophoresis system (Bio-Rad, Hercules, CA). The IPG strips were placed on top of 12.5% polyacrylamide gels and sealed with a solution of 1% (w/v) agarose containing a trace of bromophenol blue. The vertical gels were run at 10 mA per gel for 30 min followed by 25 mA per gel until the bromophenol blue had migrated to the bottom of the gel. The temperature was maintained at 15 °C using MultiTemp III system (GE Healthcare). Proteins were visualized using SYPRO Ruby Protein Gel Stain (Invitrogen, Carlsbad, CA). The SYPRO Ruby-stained gels were scanned at 532 nm using a Typhoon 9400 scanner (GE Healthcare).

### Experimental design, image analysis, and statistics

For each transformant, namely Yap1p-overexpressing transformant and control transformant, 2-D gels were run in triplicate. Additionally, a master 2-D gel was prepared, which contained a 1:1 mixture of the protein extract from the two yeast transformants. That gel, which should contain all protein spots present on the 2-D gels with samples from the Yap1p-overexpressing and the control transformant, was used during image analysis as a master gel. Image analysis was performed using the ImageMaster II software (GE Healthcare). The quantitative and statistical analyses were performed using suitable functions within the ImageMaster II software and Excel software (Microsoft, Redmond, WA). The normalized intensity of spots on three replicate 2-D gels was averaged and the standard deviation was calculated. The relative change in protein abundance for the Yap1p-overexpressing transformant (Y) *versus* the control transformant (C) (indicated with “fold change Y/C”) for each protein spot was calculated by dividing the averaged spot quantity from gels with samples from the Yap1p-overexpressing transformant by the averaged spot quantity from gels with samples from the control transformant. A two-tailed non-paired Student’s *t*-test was performed to determine if the relative change was statistically significant.

### In-gel tryptic digestion

Protein spots of interest were picked from the 2-D gels using an Ettan Spotpicking Station (GE Healthcare) and destained three times using a fresh solution of 20 mM ammonium bicarbonate containing 35% (v/v) acetonitrile (ACN). Subsequently, the gel pieces were dried by two washes using 100% neat acetonitrile and re-hydrated on ice using a solution of sequencing grade modified trypsin (Promega, Madison, WI) in 20 mM ammonium bicarbonate. The trypsin concentration depended on the intensity of the spots and was 2 to 3 ng/µl. The re-hydrated gel samples were incubated in 37 °C for overnight digestion and either analyzed immediately or stored at −20 °C until further analysis.

### Mass spectrometry

MALDI-MS spectra for peptides were acquired using a Voyager DE-STR mass spectrometer (AB SCIEX, Framingham, MA, USA) as described by Yao et al. [44]. LC-MS/MS combined with ESI-ion-trap MS was performed using an HCT-Ultra ETD II mass spectrometer from Bruker (Bremen, Germany) linked to an Easy-nLC system from Proxeon (Odense, Denmark). Spectra were acquired using the enhanced scanning mode covering a mass range from *m/z* 350 to *m/z* 1300. The LC separation of peptides was performed using a 5 µm C18 column (375 μm OD/75 μm ID × 10 cm) from NanoSeparations (Nieuwkoop, The Netherlands) and a 30 min gradient ranging from 0 to 60 percent of acetonitrile. The flow rate was 300 nl min^-1^. Data processing was performed using the Data Analysis software (4.0 SP4) (Bruker, Bremen, Germany) using default setting for processing and AutoMSn detection of compounds.

### Protein identification

Database searches using the peak list files (mgf-format) of the processed mass spectra were performed using an in-house license of Mascot (http://www.matrixscience.com), and searches were performed using the Swiss-Prot or NCBInr database. As for MALDI-MS spectra, a mass error of 50 ppm and one missed cleavage sites were permitted. In addition, variable modifications allowed included methionine oxidation and carbamidomethylation of cysteine residues. As for LC-MS/MS data a mass error of 0.3 Da was allowed for both the MS and MS/MS mode and variable modifications were set as for the database searches with the MALDI-MS data.

## Competing interests

The authors declare that they have no competing interests.

## Authors’ contributions

HJ carried out the cultivation of the yeast, the sample preparation and the 2-DE analyses, and drafted the manuscript. TK carried out in-gel digestions and mass- spectrometric analyses. LJJ conceived and organized the study and helped to draft the manuscript. All authors read and approved the final manuscript.

## Supplementary Material

Additional file 1 Spots 1 to 78. Click here for file

Additional file 2 1-5_MALDI_MS. Click here for file

Additional file 3 6-10_MALDI_MS. Click here for file

Additional file 4 11-15_MALDI_MS. Click here for file

Additional file 5 16-20_MALDI_MS. Click here for file

Additional file 6 21-25_MALDI_MS. Click here for file

Additional file 7 26-30_MALDI_MS. Click here for file

Additional file 8 31-35_MALDI_MS. Click here for file

Additional file 9 36-40_MALDI_MS. Click here for file

Additional file 10 41-45_MALDI_MS. Click here for file

Additional file 11 46-50_MALDI_MS. Click here for file

Additional file 12 51-55_MALDI_MS. Click here for file

Additional file 13 56-60_MALDI_MS. Click here for file

Additional file 14 61-65_MALDI_MS. Click here for file

Additional file 15 66-69_MALDI_MS. Click here for file

Additional file 16 70-73_MALDI_MS. Click here for file

Additional file 17 74_LC_MS_MS. Click here for file

Additional file 18 75_LC_MS_MS. Click here for file

Additional file 19 76_LC_MS_MS. Click here for file

Additional file 20 77_LC_MS_MS. Click here for file

Additional file 21 78_LC_MS_MS. Click here for file
