# Supplementary material for: Comparative proteome analysis of Saccharomyces cerevisiae: A global overview of in vivo targets of the yeast activator protein 1
Source: BMC Genomics. 2012 Jun 9;13:230. doi: 10.1186/1471-2164-13-230 (PMC3476450; doi:10.1186/1471-2164-13-230)
Supplement: Additional file 1 — Spots 1 to 78. [file 1471-2164-13-230-S1.pdf]

Spot # in MS Datasets	Working name (spot on the gel)	Protein	Description	ORF	Mascot score	95% Confidence threshold	Expectation value	Precursor mass [Da]	Peptides matched	Sequence coverage [%]
1	61	Fba1p	Fructose-bisphosphate aldolase	YKL060C	145	51	2.30E-11	39596	12 of 21	31.5
2	127	Fba1p	Fructose-bisphosphate aldolase	YKL060C	61	51	0.0056	39596	7 of 22	22.3
3	24	Pgi1p	Glucose-6-phosphate isomerase	YBR196C	125	51	2.30E-09	61261	17 of 40	35.2
4	96	Pgi1p	Glucose-6-phosphate isomerase	YBR196C	80	51	7.60E-05	61261	12 of 36	15.9
5	119	Hxk2p	Hexokinase-2	YGL253W	144	51	2.90E-11	53908	18 of 46	47.5
6	81	Tdh1p	Glyceraldehyde-3-phosphate dehydrogenase 1	YJL052W	80	51	6.60E-05	35728	7 of 18	27.1
7	80	Tdh2p	Glyceraldehyde-3-phosphate dehydrogenase 2	YJR009C	93	51	3.90E-06	35824	8 of 19	28.3
8	72	Tdh3p	Glyceraldehyde-3-phosphate dehydrogenase 3	YGR192C	89	51	9.30E-06	35724	7 of 15	33.4
9	45	Pgk1p	Phosphoglycerate kinase	YCR012W	180	51	7.20E-15	44711	15 of 24	49.5
10	65	Pgk1p	Phosphoglycerate kinase	YCR012W	97	51	1.40E-06	44711	9 of 16	27.9
11	92	Gpm1p	Phosphoglycerate mutase 1	YKL152C	178	51	1.10E-14	27592	15 of 29	44.5
12	34	Eno2p	Enolase 2	YHR174W	218	51	1.10E-18	46885	22 of 49	44.6
13	35	Eno2p	Enolase 2	YHR174W	204	51	2.90E-17	46885	20 of 30	56.1
14	38	Eno2p	Enolase 2	YHR174W	178	51	1.10E-14	46885	18 of 41	55.1
15	71	Eno2p	Enolase 2	YHR174W	124	51	2.90E-09	46885	10 of 17	23.3
16	76	Eno2p	Enolase 2	YHR174W	118	51	1.10E-08	46885	12 of 26	24.9
17	78	Eno2p	Enolase 2	YHR174W	126	51	1.80E-09	46885	16 of 32	32.3
18	16	Cdc19p	Pyruvate kinase 1	YAL038W	113	51	3.60E-08	54510	10 of 18	22.4
19	41	Cdc19p	Pyruvate kinase 1	YAL038W	175	51	2.30E-14	54510	19 of 45	31.2
20	99	Cdc19p	Pyruvate kinase 1	YAL038W	144	51	2.90E-11	54510	15 of 27	25.8
21	18	Pdc1p	Pyruvate decarboxylase isozyme 1	YLR044C	92	51	4.10E-06	61457	10 of 25	25
22	19	Pdc1p	Pyruvate decarboxylase isozyme 1	YLR044C	100	51	7.30E-07	61457	16 of 46	38.2
23	21	Pdc1p	Pyruvate decarboxylase isozyme 1	YLR044C	62	51	0.0046	61457	6 of 13	14.9
24	36	Pdc1p	Pyruvate decarboxylase isozyme 1	YLR044C	82	51	4.60E-05	61457	9 of 23	20.2
25	46	Pdc1p	Pyruvate decarboxylase isozyme 1	YLR044C	106	51	1.80E-07	61457	11 of 25	22.2
26	49	Pdc1p	Pyruvate decarboxylase isozyme 1	YLR044C	76	51	0.00019	61457	11 of 30	22.9
27	52	Adh1p	Alcohol dehydrogenase 1	YOL086C	104	51	2.90E-07	36826	9 of 21	26.1
28	115	Ald6p	Magnesium-activated aldehyde dehydrogenase	YPL061W	127	51	1.40E-09	54380	17 of 41	34.6
29	25	Dld3p	D-lactate dehydrogenase 3	YEL071W	81	51	6.00E-05	55190	7 of 12	16.7
30	11	Tkl1p	Transketolase 1	YPR074C	151	51	5.70E-12	73760	14 of 22	20.3
31	14	Tkl1p	Transketolase 1	YPR074C	92	51	5.00E-06	73760	18 of 64	22.1
32	32	Gnd1p	6-phosphogluconate dehydrogenase 1	YHR183W	76	51	0.0002	53509	8 of 21	16.2
33	30	Shm2p	Serine hydroxymethyltransferase, cytosolic	YLR058C	100	51	7.20E-07	52186	10 of 18	20.9
34	117	Aro9p	Aromatic amino acid aminotransferase 2	YHR137W	53	51	0.033	58490	8 of 28	18.3
35	31	Lys9p	Saccharopine dehydrogenase	YNR050C	74	51	0.00029	48887	7 of 17	15.5
36	4	Eft1p	Elongation factor 2	YOR133W	92	51	5.00E-06	93230	14 of 27	17.9
37	34	Eft1p	Elongation factor 2	YOR133W	77	51	0.00015	93230	14 of 49	20
38	2	Yef3p	Elongation factor 3A	YLR249W	186	70	0.00032	115872	32 of 63	33.2
39	5	Gus1p	Glutamyl-tRNA synthetase	YGL245W	192	70	3.30E-14	80972	20 of 31	29.8

40	44	Tef4p	Elongation factor 1-gamma 2	YKL081W	62	51	0.0051	46491	7 of 19	16.5
41	97	Rps7ap	40S ribosomal protein S7-A	YOR096W	90	51	7.40E-06	21609	7 of 15	18.9
42	109	Grs1p	Glycyl-tRNA synthetase 1	YBR121C	109	70	6.60E-06	75364	17 of 47	29.5
43	7	Kar2p	78 kDa glucose-regulated protein homolog	YJL034W	134	51	2.90E-10	74422	12 of 18	19.6
44	83	Rpl5p	60S ribosomal protein L5	YPL131W	102	51	4.60E-07	33694	11 of 35	43
45	77	Rpp0p	60S acidic ribosomal protein P0	YLR340W	104	51	2.90E-07	33696	8 of 16	25.6
46	89	Rps3p	40S ribosomal protein S3	YNL178W	124	51	2.90E-09	26486	10 of 20	58
47	111	Pab1p	Polyadenylate-binding protein	YER165W	147	51	1.10E-09	64304	18 of 42	37
48	112	Pab1p	Polyadenylate-binding protein	YER165W	190	51	5.30E-14	64304	21 of 41	39.9
49	9	Ssa1p	Heat shock protein	YAL005C	143	51	3.60E-11	69615	18 of 44	32.4
50	8	Ssa2p	Heat shock protein	YLL024C	130	51	7.20E-10	69427	12 of 20	23.8
51	120	Ssa2p	Heat shock protein	YLL024C	133	51	3.60E-10	69427	16 of 31	31.6
52	12	Ssb1p	Heat shock protein	YDL229W	183	51	3.60E-15	66561	16 of 24	29
53	122	Ssb1p	Heat shock protein	YDL229W	100	51	7.30E-07	66561	15 of 42	33.6
54	13	Ssb2p	Heat shock protein	YNL209W	212	51	4.60E-18	66554	20 of 35	40.3
55	106	Hsp82p	ATP-dependent molecular chaperone	YPL240C	222	70	3.30E-17	81356	23 of 38	36.7
56	3	Hsc82p	ATP-dependent molecular chaperone	YMR186W	183	51	3.60E-15	80850	20 of 35	29.1
57	107	Sse1p	Heat shock protein homolog	YPL106C	109	70	6.60E-06	77318	14 of 39	24.5
58	98	Tsa1p	Peroxiredoxin TSA1	YML028W	72	51	0.00048	21576	6 of 24	30.1
59	50	Oye2p	NADPH dehydrogenase 2	YHR179W	206	51	1.80E-17	44983	17 of 30	43.8
60	51	Oye2p	NADPH dehydrogenase 2	YHR179W	108	51	1.10E-07	44983	9 of 18	28
61	B18	Atp2p	ATP synthase subunit beta	YJR121W	146	51	1.90E-11	54760	17 of 38	48
62	23	Bfr1p	Nuclear segregation protein	YOR198C	106	51	1.80E-07	54606	12 of 33	27.2
63	67	Stm1p	Suppressor protein	YLR150W	77	51	0.00013	29977	9 of 46	34.4
64	105	Cdc48p	Cell division control protein 48	YDL126C	212	70	3.30E-14	91939	21 of 27	29.6
65	108	Cdc48p	Cell division control protein 48	YDL126C	103	70	2.60E-05	91939	9 of 12	14.4
66	113	Vma1p	V-type proton ATPase subunit A	YDL185W	111	51	4.20E-06	118562	23 of 60	21.9
67	20	Vma2p	V-type proton ATPase subunit B	YBR127C	60	51	0.0066	57713	8 of 32	23.6
68	73	Sis1p	Protein SIS1	YNL007C	92	51	4.30E-06	37567	9 of 21	25.6
69	60	Rnr4p	Ribonucleoside-diphosphate reductase small chain 2	YGR180C	92	51	4.90E-06	40029	11 of 24	31
70	87	Bgl2p	Glucan 1,3-beta-glucosidase	YGR282C	60	51	0.0068	34097	7 of 25	25.6
71	124	Bmh1p	Protein BMH1	YER177W	122	51	4.60E-09	30073	14 of 35	43.8
72	126	Ola1p	Uncharacterized GTP-binding protein	YBR025C	102	51	4.60E-07	44146	9 of 14	30.2
73	62	Ynn4p	Uncharacterized protein YNL134C	YNL134C	67	51	0.0015	41139	8 of 26	30.9

		LC-MS/MS Protein	Description	ORF	Mascot score	Precursor mass [Da]	Peptides matched	Sequence coverage [%]
74	S17	Srp1p	Importin subunit alpha	YNL189W	329	60404	11	8
75	S37	Sgt2p	Small glutamine-rich tetratricopeptide repeat-containing protein 2	YOR007C	1048	37195	62	55
76	S11	Pdi1p	Protein disulfide-isomerase	YCL043C	643	58191	23	11
77	S19	Pdi1p	Protein disulfide-isomerase	YCL043C	183	58191	7	6
78	S30	Pdi1p	Protein disulfide-isomerase	YCL043C	212	58191	10	7
